# Irrigation Water Quality for Leafy Crops: A Perspective of Risks and Potential Solutions

**DOI:** 10.3390/ijerph120707457

**Published:** 2015-07-03

**Authors:** Ana Allende, James Monaghan

**Affiliations:** 1 Research Group on Quality, Safety and Bioactivity of Plant Foods, Department of Food Science and Technology, CEBAS-CSIC, Campus Universitario de Espinardo, 30100 Murcia, Spain; E-Mail: aallende@cebas.csic.es; 2 Fresh Produce Research Centre, Department of Crop and Environment Sciences, Harper Adams University, Newport, Shropshire, TF10 8NB, UK

**Keywords:** leafy vegetables, irrigation water, food safety, QAS, GAP, water disinfection treatment, hydroponics

## Abstract

There is increasing evidence of the contribution of irrigation water in the contamination of produce leading to subsequent outbreaks of foodborne illness. This is a particular risk in the production of leafy vegetables that will be eaten raw without cooking. Retailers selling leafy vegetables are increasingly targeting zero-risk production systems and the associated requirements for irrigation water quality have become more stringent in regulations and quality assurance schemes (QAS) followed by growers. Growers can identify water sources that are contaminated with potential pathogens through a monitoring regime and only use water free of pathogens, but the low prevalence of pathogens makes the use of faecal indicators, particularly *E. coli*, a more practical approach. Where growers have to utilise water sources of moderate quality, they can reduce the risk of contamination of the edible portion of the crop (*i.e.*, the leaves) by treating irrigation water before use through physical or chemical disinfection systems, or avoid contact between the leaves and irrigation water through the use of drip or furrow irrigation, or the use of hydroponic growing systems. This study gives an overview of the main problems in the production of leafy vegetables associated with irrigation water, including microbial risk and difficulties in water monitoring, compliance with evolving regulations and quality standards, and summarises the current alternatives available for growers to reduce microbial risks.

## 1. Introduction

Water of inadequate quality has the potential to be a direct source of contamination and a vehicle for spreading localized contamination in the field, facility or transportation environments in the production of fresh produce crops [1]. A number of comprehensive review articles have been published which highlight irrigation water as a source of pathogenic microorganisms in produce [2,3,4,5,6,7,8]. Pathogenic microorganisms associated with irrigation water include bacteria, viruses and parasites (protozoa and helminths). Although viruses and parasites are of paramount importance and can be transmitted to fresh produce via irrigation water, this paper focuses on pathogenic bacteria.

The increasing evidence of contamination of produce from irrigation water has been reviewed recently by Uyttendaele *et al.* [9] and contamination events were identified where water is a risk factor in the production and harvesting of fresh produce. However, the authors made clear that identification of the implicated food vehicle and/or the location of the point of food contamination in fresh produce-associated outbreaks are a recurrent challenge. 

Despite this evidence, research focused on microbial quality of agricultural water is relatively limited. Most research regarding microbial quality of water (e.g., pathogen prevalence or indicator organisms) has been conducted for objectives related to reclaimed water, drinking and recreational water supplies and the effects of agriculture on the environment [10]. In 2011, Pachepsky *et al.* [5] highlighted that no databases on microbial quality of irrigation water had been compiled. However, there is an increasing number of recent research papers that focus on the evaluation of the microbial quality of irrigation water used for the production of fresh produce and its significance as a source of contamination e.g., [11,12,13,14,15,16,17,18,19,20]. Several of these published papers show the results obtained within the frame of a research project consortium of the FP7 project Veg-i-Trade (www.vegitrade.org) focused on the impact of climate change and globalisation on safety of fresh produce. 

Growers use a variety of water sources for field operations and irrigation and much knowledge is needed to relate risk factors associated with the transfer coefficients for pathogens by source, concentration and use [10]. Quantitative microbial risk assessment (QMRA) is being applied to establish the links between concentrations of pathogenic microorganisms in agricultural water and the probability of illness [21,22,23]. Lately, as a result of several longitudinal microbiological surveys, different scenarios have been evaluated to estimate the potential impact of agricultural practices, including water treatments and the application of potable water, on the *E. coli* levels and prevalence of pathogenic microorganisms in fresh produce [24,25].

Based on this evidence, standards and guidelines for Quality Assurance Schemes (QAS) have been designed to cultivate prevention and control of food safety hazards [26] but QAS have become not only a prevention strategy but also a marketing strategy used by the industry, which is pushing towards zero-risk for many factors including irrigation water sources, in response to consumers concerns over food safety [27]. In many cases, current QAS require growers to develop and implement management systems and to risk assess the irrigation water sources to reduce microbial risks. Increasingly QAS are requiring stringent testing regimes of water coming into contact with the edible portion of leafy vegetables [28]. However, some studies highlight that the required monitoring programs can be self-defeating and create additional uncompensated monitoring costs [26]. 

Growers should also consider other alternatives to guarantee the safety of the produce they produced under any circumstance. Surface water represents one of the most risky water sources, thus where growers are utilising surface water for agricultural applications, particularly for leafy greens or produce that is intended to be consumed raw, and mitigation strategies may be required [29]. Several strategies have been proposed to reduce the risk of produce contamination with pathogens during irrigation [5]. The main aims of these strategies is to reduce the use of uncontrolled sources of water and to establish distance limits for water resources used for irrigation from livestock housing, stored effluents, and/or land spread with manure [30]. 

Water treatment is also a feasible strategy to guarantee the microbial quality of irrigation water. Treating water during storage and while in the delivery systems may represent a good alternative to high frequency microbial testing [8] and probably the only possibility for growers using irrigation water with microbial loads above regulatory thresholds. Another option for growers using water sources of poor microbiological quality is the use of different production systems, such as hydroponic systems and drip irrigation, which avoid contact between the edible part of the crop and the irrigation water. 

This study gives an overview of the main problems associated with irrigation water, including microbial risk and difficulties in water monitoring, compliance with evolving regulations and quality standards, and summarises the current alternatives available for growers to reduce microbial risks.

## 2. Irrigation Water as a Risk Factor

Agricultural water has been defined as a major risk factor in the contamination of leafy crops eaten raw as salads [30]. When available, fresh water is consumed for agricultural production but water scarcity is becoming a major threat to the sustainability of agriculture, which needs to rely much more on marginal water sources, including treated wastewater [31]. However, little is known regarding the microbial quality of irrigation water used for leafy crop production. Systematic sampling focused on determining the risks of microbial contamination associated with different water sources are rare and most of the existing information comes from U.S. The available literature indicates that generic *E. coli* levels and prevalence of pathogenic foodborne bacteria in irrigation water significantly varies depending on several factors including seasonality, geographical location and weather conditions among others (reviewed by [8,9]). The Center for Produce Safety [10] have published a report to provide a summary of the scientific and technical information related to factors that affect the microbiological safety of agricultural water. This report summarizes the most recent research on irrigation water and gives a good overview of the needs for future research including sampling strategies that provide an estimate of the true underlying distribution of bacteria in a water system, correlation of field and water system management practices with pathogen prevalence in agricultural water samples, as well as a better understanding of risk factors leading to survival and/or growth of pathogens on fresh produce following application of contaminated water.

**Table 1:** Enumeration of microbial indicators and prevalence of foodborne pathogens in water used to irrigate fresh produce in Europe.

Country	Produce	Water Source	Microorganisms	Average cfu/100 mL	Prevalence	Reference
Belgium	Strawberry	Groundwater	STEC	−	0/22	Delbeke *et al.*, 2015
			*E. coli* spp.	1	4/22	
		Rainfall water collected in ponds	STEC	−	11/56	
			*E. coli* spp.	40–45	40/56	
Belgium	Lettuce	Rainfall water collected in open wells and bore hole water	STEC	−	6/68	Holvoet *et al.*, 2014
			*Campylobacter* spp.	−	37/120	
			*Salmonella* spp.	−	1/68	
			*E. coli* spp.	30–35	90/120	
Spain	Baby spinach	Surface water collected in water reservoirs	STEC	−	0/50	Castro-Ibañez *et al.*, 2015
			*Salmonella* spp.	−	1/50	
			*E. coli* spp.	5–10	72/250	
Spain	Tomatoes	Surface water	STEC	−	0/16	López-Gálvez *et al.*, 2014
			*Salmonella* spp.	−	1/16	
			*E. coli* spp.	20–25	6/32	
			*Listeria* spp.	30–35	26/30	
		Reclaimed water	STEC	−	0/16	
			*Salmonella* spp.	−	2/16	
			*E. coli* spp.	240–280	31/32	
			*Listeria* spp.	350–400	26/30	
Italy	Tomatoes	Tap water	*E. coli* spp.	−	0/30	Forslund *et al.*, 2012
		Reclaimed water	*E. coli* spp.	10,300	11/30	
Crete	Tomatoes	Tap water	*E. coli* spp.	400	2/31	
		Reclaimed water	*E. coli* spp.	596	4/31	

**Table 2:** Characteristics, advantages and disadvantages of water treatment technologies for irrigation water.

Water Treatment	Active Agent	Recommended Dose	Reported Microbial Reductions Range (Log cfu/mL)	Advantages	Disadvantages
Sodium hypochlorite	Hypochlorous acid	2–5 mg/L	0.2–4.0	High bactericidal action Liquid ready to use Low operating costs	Organic matter reduces its efficacy Influenced by pH Storage of large volumes By-product formation (trihalomethanes and chlorates among others)
Calcium hypochlorite	Hypochlorous acid	2–5 mg/L	0.2–4.0	High bactericidal action Tablets ready to use Low operating costs	Organic matter reduces its efficacy Influenced by pH By-product formation (trihalomethanes and chlorates among others)
Chlorine dioxide	Chlorine dioxide molecule	0.1–5.0 mg/L	0.5–5.0	High bactericidal action Effective at a wide pH range (4–10) Does not react with organic matter as chlorine	“*in situ*” generation or use of stabilized solutions By-product formation (mostly chlorates)
Ultrasound	Cavitation	20–40 kHz	3.0	Not affected by pH Easy to use No formation of disinfection by-products	Lack of residual bactericidal action
UV-C	DNA damage	1–200 mJ/cm^2^	0.5–5.0	High bactericidal action Not affected by pH Easy to use No formation of disinfection by-products Low operational costs	Water turbidity affects efficacy Lack of residual bactericidal action
Membrane filtration	Particle interception		1.0–5.0	Not affected by pH Easy to use No formation of disinfection by-products	Filter blockage

### Current Situation in Europe

Recently, a comprehensive review has summarized the main irrigation water sources used in Europe highlighting municipal water, rainwater, groundwater and surface water as the most relevant [9]. In a temperate production area, such as the UK, the primary sources of water for irrigation are reported to be surface water (54%) and groundwater (41%), with the remainder coming from public mains water, rainwater and other sources [32,33]. However, in arid and semi-arid parts of Europe, such as the South of Spain, Italy and Greece, which are confronting increasing water shortages, treated municipal wastewater is a valuable water source for recycling and reuse in agriculture. As an example, 347 hm^3^ of treated wastewater were reused in 2010 in Spain, particularly in the South East of Spain where almost 60% of the reclaimed water produced was reused [34] and 233 hm^3^ in Italy [35]. Although the use of untreated wastewater for crop production is not a recommended practice, research results indicate that tertiary water treatment, including final disinfection using UV light, chlorination and/or ultrasound have been shown to be effective in removal of indicator microorganisms and pathogens to below limits of detection [5,36]. 

**Figure 1:**
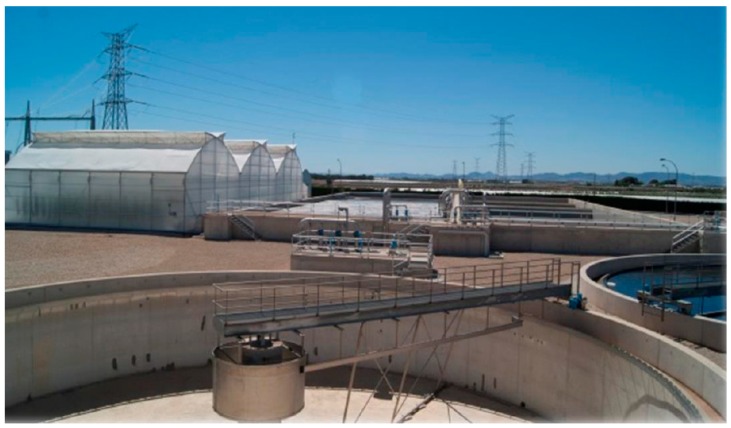
A secondary treatment wastewater plant located in a greenhouse production unit to provide irrigation water for tomato production. Reprinted with permission of Quality and Safety Lab CEBAS-CSIC.

Research related to microbial quality of agricultural water in Europe is limited, although recent research studies have highlighted the microbial risks associated with different water sources (Table 1). In most cases, longitudinal microbiological surveys have been carried out to evaluate the microbiological quality of produce samples, excluding the monitoring of risk factors such as irrigation water. It should be taken into account that the performance of irrigation water-quality monitoring and management practices are highly variable within countries as well as among different countries, with large differences across Europe. In 2006, Tyrrel *et al.* [37] reported that in UK, the majority of growers do not irrigate with water that would conform the European Union Drinking Water Standard [38] and used water that was faecally contaminated, but that typical faecal coliform concentrations were ≤1000/100 mL. More recently, Holvoet *et al.* [16] monitored eight Belgian lettuce farms to establish the relationships between levels of indicator bacteria and detection of enteric zoonotic pathogens. A high prevalence (75%, *n =* 120) of *E. coli* was found in the irrigation water sources with 65% of the positive samples having *E. coli* levels ≥1 log cfu/100 mL while 26% of the samples showed *E. coli* counts ≥2 log cfu/100 mL, which is above most of the irrigation water-quality standards. Additionally, 35% of the collected samples were positive for at least one pathogen (*Salmonella*, *Campylobacter* or Shiga toxin-producing *E. coli* (STEC)). In this study, most of the monitored farms used open wells to hold collected rainfall water. Previously reported *E. coli* values for surface water collected from rivers within an agricultural landscape were within the range 1.5–3.3 log cfu/mL [39], confirming the evidence that surface water, including rivers, streams, and creeks have unpredictable water quality and activities upstream can rapidly change the levels of contaminants [9]. A study focused on Belgian strawberry production reported significant differences in the microbiological quality of irrigation water obtained from different water sources including groundwater and collected rainfall water stored in ponds [19]. They reported that water obtained from the ponds was positive for *E. coli* (40/56) with an average level of 1.6 log cfu/100 mL and almost 20% of these samples were positive for STEC. However, groundwater samples showed much lower levels of *E. coli* and these samples did not contain STEC. Water stored in reservoirs or lagoons provides both an opportunity for pathogen die-off through natural UV but also for new contamination associated with the use of reservoirs as wildlife habitats [37]. In many European countries, a significant proportion of irrigation water is abstracted and stored in farm reservoirs. Castro-Ibañez *et al.* [17] monitored several water reservoirs used to irrigate growing fields of leafy greens. Samples were analysed for presence of foodborne pathogens and only one sample was positive for *Salmonella* spp. Reported *E. coli* prevalence and concentration in irrigation water samples from water reservoirs was lower than that reported from open wells in Belgium. Observed differences between Belgium and Spain might be due to differences in weather conditions as solar radiation has been highlighted as an important mechanism for bacterial decline in environmental samples including irrigation water [18]. Urban wastewater has been used in agriculture as a way to overcome water scarcity in the South of Europe. The European Water Framework Directive [40], specifies that treated wastewater should be used in agriculture where and whenever possible. Currently, urban wastewater is mainly used for irrigation in combination with production systems that avoid direct contact between the water and the edible part of the fresh produce. In countries such as Spain, Greece and Italy, commercial production using urban wastewater as irrigation water usually involves production of tomatoes, peppers and cucumbers grown hydroponically in greenhouses. Sometimes, wastewater treatment plants, consisting of a train of individual unit processes, are located close to the greenhouse (Figure 1). 

Studies focusing on the microbial quality of reclaimed water used for irrigation reported presence of faecal contamination within the range 2–4 log *E. coli* cfu/100 mL and also presence of pathogenic microorganisms such as *Salmonella* spp. [41,42] (Table 1). Production systems that minimize irrigation water contact with the edible portion of the crop seem to reduce the risk of contamination. *Codex Alimentarius* [43] reported that plants grown in hydroponic systems absorb nutrients and water at varying rates, constantly changing the composition of the re-circulated nutrient solution and because of this water used in hydroponic culture should be changed frequently, or if recycled, should be treated to minimize microbial and chemical contamination.

## 3. Regulations, Guidelines and Microbial Quality Standards for Water Used in Primary Production

The microbial quality of irrigation water has been related with food safety for more than 25 years. In Europe, specific microbial criteria are only currently established in guidelines and quality assurance standards (QAS), but specific microbial criteria have been introduced in U.S. legislation and these are starting to have an impact on European standards.

### 3.1. Legislation—The European Commission

Regulation (EC) No 852/2004 on the hygiene of foodstuffs defines potable water as “*meeting the minimum requirements laid down in Council Directive 98/83/EC* [38] *on the quality of water intended for human consumption*”, clean water is defined in Regulation (EC) No. 852/2004 [44], as “*clean seawater and fresh water of a similar quality*” and clean seawater is defined as “*natural, artificial or purified seawater or brackish water that does not contain micro-organisms, harmful substances or toxic marine plankton in quantities capable of directly or indirectly affecting the health quality of food*” [44]. Specific microbiological criteria are not defined but growers must be able to demonstrate that their operations are managed in a way that controls food safety risks, including those associated with the use of water.

### 3.2. Guidelines

#### 3.2.1. World Health Organization (WHO)

Whilst it was clear that care was needed in the safety of water used to irrigate crops that are eaten raw, such as leafy salads; prior to 1973 there was no generally accepted standard of microbial water quality. In 1973 the World Health Organization (WHO) addressed the issue that drinking water microbial quality standards (*i.e.*, ≤2.2 cfu/100 mL coliforms) were not a realistic target and produced a guideline value of ≤100 cfu/100 mL coliforms for unrestricted irrigation water (*i.e.*, water used to irrigate crops that will be eaten uncooked) derived from wastewater [45]. Following consideration of what was achievable by wastewater treatment processes and associated epidemiological studies of wastewater use, these guidelines were revised in 1989 to a geometric mean of ≤1000 cfu/100 mL faecal coliforms and an arithmetic mean of ≤1/L intestinal nematode during the irrigation period [46]. These standards have been criticised, particularly from those developing guidelines with the aim for “zero-risk” irrigation water [47]. The WHO has more recently changed the approach and there are now no definitive values for microbiological guidelines for irrigation water. Instead, irrigation water safety should be based upon risk assessment as recommended in WHO documents and water guidelines in advanced economies should rely on in-country standards [48,49].

#### 3.2.2. *Codex Alimentarius* Commission

Baseline guidance on safety requirements of irrigation water is provided through *Codex Alimentarius:* both the General Principles of Food Hygiene-CAC/RCP 1–1969 [50] and the Code of hygienic practice for fresh fruit and vegetables-CAC/RCP 53-2003 [42] address the issue. However this only provides a “*general framework of recommendations […] rather than providing detailed recommendations for specific agricultural practices…”* [43] *i.e.*, the water used in primary production must be safe. However the microbial requirements are not defined. For example, in the Leafy Green annex of the Code of hygienic practice for fresh fruit and vegetables-CAC/RCP 53–2003 growers are required to “*seek appropriate guidance on water quality and delivery methods to minimize the potential for contamination with microbial pathogens*” [43]. And where water comes in to “*substantial contact with the edible portion of the leafy vegetable should meet the standards for potable or clean water*” [43] where these are defined as: *Potable water–*water which meets the quality standards of drinking water such as described in the WHO Guidelines for Drinking Water Quality; *Clean water–*water that does not compromise food safety in the circumstances of its use [43].

### 3.3. Microbial Quality Standards for Water Used in Primary Production

#### 3.3.1. QAS—1st Generation

In the early 1990s the UK fresh produce supply chain started to develop Quality Assurance Schemes (QAS) also known as Retailer Codes of Practice partly in response to the 1990 UK Food Safety Act that required growers to be treated as running food businesses within a consolidated supply and as a consequence growers were exposed to due diligence imposed from the retail end of the supply chain [51,52]. Amongst these QAS were Assured Produce–1991; Tesco Natures Choice–1992 and, more widely relevant, EurepGAP–1997 [53]. In essence these QAS followed Codex guidelines and required systems to be in place to ensure that the water used in production was safe. The approach followed Hazard Analysis and Critical Control Point (HACCP) principles such as to risk assess water sources and use but did not define water quality criteria. In 2004 Marks and Spencer (M&S) released a new QAS “Field to Fork” that required growers to test their irrigation water for *E. coli*, but again no criteria were stipulated as to what an acceptable level of indicator organism would be [53].

#### 3.3.2. QAS—2nd Generation

The QAS increased their focus on microbial risks associated with irrigation water and a new generation of schemes (e.g., Red Tractor Fresh Produce Scheme, GlobalGAP, Tesco Nurture, M&S Field to Fork version 2) was developed in the 2000s [51]. As with the first generation of schemes, growers were still required to develop and implement management systems along HACCP principles and to risk assess all water sources. There was also more guidance on risk assessment methodology but critical levels of indicator organisms (with some confusion between *E. coli* and faecal coliforms) in water used in primary production were now defined. Generally, the criteria were based on the WHO guidance for water for irrigation of produce that can be consumed uncooked of ≤1000 faecal coliforms cfu/100 mL with an additional reporting level of ≥400 cfu/100 mL in Tesco Nurture and M&S Field to Fork version 2, which has become more stringent in later revisions for leafy crops. 

#### 3.3.3. QAS—3rd Generation

In 2011 the Food Safety Modernization Act (FSMA) became US law [54]. The FSMA proposed microbial quality standards for irrigation water and wash water that came into contact with the edible portion of fresh produce crops. Although the original microbial quality standards are being revised to include rules on produce safety that are more flexible and less burdensome in key areas [55], these standards have been applied in Good Agricultural Practices (GAP) used in the USA and also more widely. One example is the McDonalds GAP [28] where the grower is required to implement systems to ensure food safety, based on HACCP principles and risk assessment as with previous generations of QAS, but clear metrics and microbial criteria are now defined. Irrigation water that may contact the edible portion of the crop must have no more than 235 cfu (or most probable number (MPN), as appropriate) generic *E. coli* per 100 mL for any single sample, or a rolling geometric mean (*n =* 5) of more than 126 cfu (or MPN, as appropriate) generic *E. coli* per 100 mL of water [28]. A pre-planting water sampling programme is needed for any irrigation water used (and stored rain water). Each water source must have a minimum of one sample analyzed from the point of application closest to the crop to establish that water quality is acceptable before being used for irrigating the crop (*i.e.*, ≤235 cfu generic *E. coli* per 100 mL). Subsequently, during crop growth, a set of at least five irrigation water samples must be collected prior to harvest to establish the rolling geometric mean. The sampling frequency is not stipulated but five samples must be taken within the crop growth period or 30 days, whichever is the shorter [28].

More recently the FDA has revised the upper limit of the irrigation water quality criteria to a statistical threshold value (*i.e.*, a value that should not be exceeded by more than 10 percent of the samples taken) ≤410 CFU of generic *E. coli* in 100 mL of water [55] and it would be anticipated that this standard would start to be used in some QAS. 

It has become clear that the industry is being pushed towards zero-risk irrigation water sources in response to supply chain concerns over food safety and that there is a greater requirement for frequent water testing regimes with complex calculations of critical values. These high standards will be difficult to achieve with open water sources such as rivers or stored water that are regularly tested, and for some producers their water sources will not comply with the requirements. This may lead to alternative strategies being required.

## 4. Solutions

### 4.1. Monitoring Pathogen and Indicator Species in Water Sources

Testing water can be used to establish a history of microbiological quality and inform a risk ranking for a water source [9]. The prohibitive cost and time requirement of pathogen detection make microbial indicators a good strategy to characterize microbial contamination in agricultural water [56]. Several studies report that indicator bacteria, and particularly generic *E. coli* concentrations, are not correlated with the presence of pathogens such as *E. coli* O157 or *Salmonella* spp. in water samples, suggesting that *E. coli* data might not be suitable to predict the risk of exposure to pathogenic strains [10,57,58]. However, a logistic regression analysis of samples taken monthly for 12 months from 18 locations throughout Central Florida showed that *E. coli* concentration can predict the probability of enumerating selected *Salmonella* levels, indicating that *E. coli* provides a reasonable way to predict *Salmonella* levels in surface water. According to this, in recent longitudinal microbial surveys carried out in Europe, presence of elevated levels of *E. coli* increased the probability of presence of pathogens (STEC and *Salmonella* spp.) [16,18]. Additionally, Wilkes *et al.* [59] reported that faecal indicators such as *E. coli* spp. were conservative surrogates for a variety of pathogenic microorganisms in surface waters within an agricultural landscape. Based on these reports, *E. coli* spp. could be identified as suitable for a hygiene criterion at primary production of leafy greens and can be applied for validation and verification studies of GAP.

However, factors such as the dynamic nature of agricultural water microbial quality, the time lag between obtaining agricultural water testing results and water use, and the fact that current water sampling strategies are based on an assumption that bacteria are floating as single cells in water, make water monitoring limited in its ability to monitor microbial risks [10]. In addition, Won *et al.* [60] reported that *n* > 5 canal and *n* > 14 reservoir samples were needed to calculate *E. coli* concentrations at a precision level of 85% with 95% confidence interval under the same environmental conditions during the testing period; a frequency much greater than required by the most stringent QAS. The frequency of testing will vary depending on the water source and the risk of environmental contamination [3]. For example, Holvoet *et al.* [61] observed in a study of leafy vegetable farms in Belgium that contamination of irrigation water was more prevalent in open-field production compared to greenhouse production. An additional limitation of the testing approach is the costs of an effective monitoring program which are usually too expensive for most small to medium growers [9]. Hence, alternatives to monitoring sampling plans such as water treatments and the selection of less risky production systems, which avoid contact between the edible part of the plant and irrigation water, may be an alternative option in reducing microbial risks to leafy vegetables. 

### 4.2. Water Treatments

Physical and chemical disinfection systems have been explored as methods to remove human pathogens from agricultural water sources (Table 2), although disinfection treatment of irrigation water is still a very limited practice [4]. Nowadays, chemical sanitizers are the most commonly used water treatments, although environmentally friendly alternatives are being demanded, particularly for organic production. In fact, concerns have risen recently regarding both the absence of water treatment and the excessive use of potentially toxic chemicals to treat irrigation water. Norton-Brandão *et al.* [35] presented a critical review of a wide range of urban reclamation technologies that could be applied to water; of these, the treatments relevant to agricultural irrigation water included coagulation, flocculation, filtration and chemical disinfection [8]. 

Among commercially available water treatments, chlorine-based sanitizer remains the most common water treatment used for the removal of biohazards from irrigation water [4,8]. The advantages of using water treatment are generally associated with microbial reductions of both foodborne pathogens as well as phytopathogens and the reduction of contamination from biofilm formation in the irrigation pipes (Table 2) [61]. There are many inexpensive commercial applications of chlorine-based sanitizers available for growers. When using this type of water disinfectants, a key step is the estimation of the peak chlorine demand over a range of typical operating conditions, where the peak chlorine demand is defined as the maximum amount of free chlorine in a batch of water that is “used up” by soil and organic materials added with product during washing [62]. Two of the more widespread treatments in Europe and also U.S., are calcium hypochlorite or chlorine dioxide but limitations of chlorine-based disinfectant in terms of formation of disinfection by-products and the potential negative effect on the environment have limited its use in agricultural water. Nevertheless, the goal of the dosing system is, in most cases, to reduce *E. coli* levels to be within a compliant range, for which a minimal dose of disinfectant is usually enough. This reduces the potential detrimental effects on the farm soil or environment from disinfection by-products [63] in the short-term but there are still concerns for chronic effects of large-scale use over long periods of time [4]. Additionally, the volumes of water that are commonly used in a medium-large production field are very high (50–100 m^3^), and the cost associated to the treatment of irrigation water can be substantial. 

**Figure 2:**
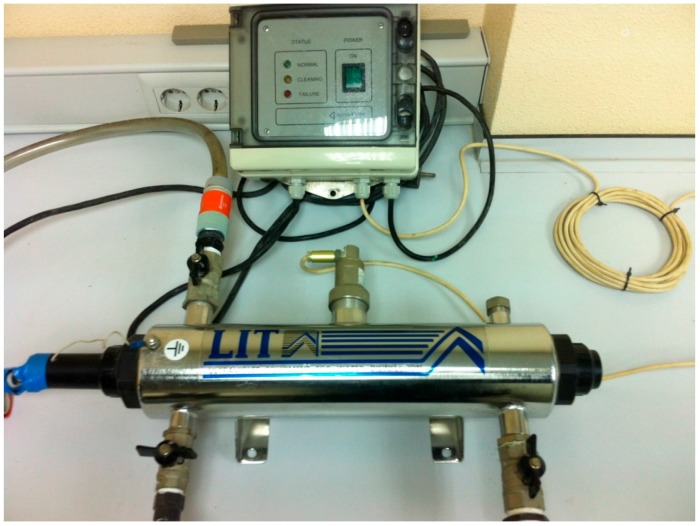
UV-C treatment system where water passes through a vessel while it is illuminated by UV-C lamps located in the vessel. Reprinted with permission of Quality and Safety Lab CEBAS-CSIC.

Greener technologies based on physical treatments such as ultrasound (US), ultraviolet light (UV-C) and filtration have been successfully tested to reduce microbial loads of irrigation water [4,29,36]. US technology has the advantage of reducing microbial loads, including algae, without the detrimental effects of the formation of disinfection by-products [36]. Other reported advantages of US include potential simultaneous oxidation, thermolysis, shear degradation, and enhanced mass transfer processes [64]. UV-C light has also been used frequently to disinfect irrigation water and requires the installation of a UV-C treatment system where water passes through a vessel while it is illuminated by UV-C lamps located in the vessel (Figure 2). These systems are usually relatively cheap and represent a good treatment option if water turbidity is low [65]. However, a pre-treatment usually based on sand filtration and also regular maintenance of the lamp are needed to ensure proper efficacy of the system. Membrane and sand filtration have been reported as effective technologies to remove pathogens from water [35]. Different pore sizes are applied for different applications. Thus, membrane filters classified as micro- (0.1–10 µm), ultra (0.002–0.1 µm), nano (0.0005–0.002 µm) filtration or reverse osmosis (<0.0005 µm) are used for removal of microorganisms while larger pore sizes and rapid sand filters are used to reduce soil and plant material that may clog the membrane filters [66]. Membrane filtration is more effective at controlling pathogens when combined in a series of multi-stage filters and in combination with other treatments that have other modes of action such as chlorine [67]. Innovative filtration systems such filters containing sand and/or materials with reactive components have been explored as potential water treatments [10]. Slow sand filtration controls microorganisms present in the water by biological, physical and chemical reactions. In these systems, microbial removal is carried out by a complex microbial community located in the upper layer on the sand bed but also by physically entrapping the pathogens and debris [67]. Biosand filter zero-valent iron incorporated (ZVI) treatments, which have been used in permeable reactive barriers to remove a broad range of chemical contaminants in groundwater, have also been reported as potential water treatments [68]. Ingram *et al.* [69] have recently proposed ZVI treatment as a cost-effective mitigation option for irrigation water to help small farmers reduce risk of foodborne *E. coli* infections associated with contamination of leafy greens. 

Whilst alternative systems are being proposed in scientific studies, the evaluation of the site-specific applicability of any of these technologies, such as maintenance costs, safety, and biological effects on crops and humans, is missing [4,5]. In general, water treatment technologies for agricultural water have not been deeply evaluated and in most of the cases, operational and maintenance costs are knowledge gaps crucial to the decision making process of farmers [4]. On the other hand, process control for water treatment technologies is mandatory to guarantee the efficacy of the treatment. A good understanding and consistent implementation of systems to monitor, control, and document water treatment performance are needed because without the process control, all water treatment systems may give a “false confidence”.

### 4.3. Alternative Production Systems

An alternative strategy to managing the risk of contaminating leafy vegetables through faecally contaminated irrigation water is to avoid direct contact with the edible parts of the crop. This can be achieved through selecting the irrigation method and/or the production system used to the crops. Application of microbial-contaminated irrigation water using subsurface drip irrigation has been shown to reduce contamination of crops including lettuce at harvest compared to furrow irrigation [70]. However, even if direct contact between irrigation water and the edible part of the leafy crop is avoided, irrigation water may contaminate the soil or substrate, where the bacteria can survive for some time [30] and irrigation or rainfall splash may contaminate the crop [11].

**Figure 3:**
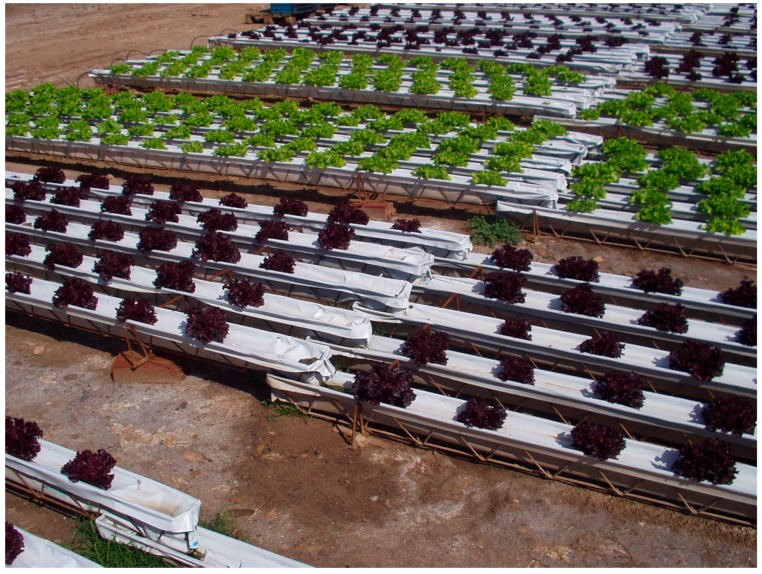
Nutrient film techniques (NFT), a type of soilless systems where a thin film of nutrient solution flows through plastic channels which contain the plant roots and laid on a slope in order to grant the constant flow of nutrient solution. Source: James M. Monaghan (Harper Adams University).

Soilless systems, such as hydroponic floating systems [71] or nutrient film techniques (NFT) [72], are being used for leafy vegetables with short production cycles allowing a better control and standardization of the cultivation process (Figure 3). Many advantages have been attributed to the use of soilless systems in greenhouses to produce leafy greens, but reductions in product quality and shelf life have been observed [73] which may limit the use of these systems. However, recent studies carried out in commercial agricultural production sites showed that the use of poor quality irrigation water combined with the use of soilless production systems considerably reduced microbial contamination risks to fresh produce [42,74]. 

This reduction can be attributed to preventing contact between the irrigation water and the edible part of the plant. In a non-commercial hydroponic system lettuce grown using diluted effluent from secondary UV treated grey-water was shown to have an acceptable level of *E. coli* spp. on the harvested leaves, even though levels on the roots were high [75]. The use of alternative production systems such as greenhouses and hydroponics are assumed to be safer than open field production from the microbiological point of view due to the minimization of some risk factors associated with sources of pre-harvest contamination, but also a greater control of water disinfection with water being recirculated and cleaned periodically [76]. The nutrient solution, and hence water, used in soilless systems is one of the most important aspects for the success of leafy greens production [77] and the quality of water can be better controlled in soilless systems [63] both from a plant pathogen and human pathogen perspective. Nevertheless, before hydroponics could be used to grow crops using lower quality water more knowledge is required on the risks posed from root contamination and internalization of bacteria (e.g., [78]) from contaminated hydroponic water sources.

## 5. Conclusions

There is an increasing evidence of contamination of produce from irrigation water, but scarce information on the microbial quality of agricultural water is available. Despite this, retailers selling leafy vegetables are risk averse and are targeting zero-risk production systems. As a consequence the requirements for irrigation water quality and safety in QAS have evolved and become more stringent. One approach is for growers to identify water sources that are contaminated with potential pathogens through a monitoring regime and only use water free of pathogens, but this approach is very costly and ultimately does not ensure safe water. The use of faecal indicators, particularly *E. coli*, allows growers to identify water sources where there is a route of faecal contamination, allowing risk categorization of water sources [9,30]. Where growers only have access to water sources of moderate quality, with low levels of faecal contamination, they have two main options available to produce leafy vegetables that will not pose unacceptable risks to the consumer: treat water before use, using physical or chemical disinfection systems, with chlorine the most commonly used system currently; or reduce/eliminate contact with the leaves from irrigation water through irrigation water placement *i.e.*, drip or furrow, or the use of soilless growing systems. It is likely that a combination of approaches will be needed to meet the microbial requirements of leafy vegetables and ensure safe food for consumers.
